# Secondary Metabolites in Edible Species: Looking beyond Nutritional Value

**DOI:** 10.3390/foods10051131

**Published:** 2021-05-19

**Authors:** Ana M. L. Seca, Antoaneta Trendafilova

**Affiliations:** 1 cE3c—Centre for Ecology, Evolution and Environmental Changes/Azorean Biodiversity Group & Faculty of Sciences and Technology, University of Azores, Rua Mãe de Deus, 9500-321 Ponta Delgada, Portugal; 2 LAQV-REQUIMTE, University of Aveiro, 3810-193 Aveiro, Portugal; 3 Institute of Organic Chemistry with Centre of Phytochemistry, Bulgarian Academy of Sciences, Acad. G. Bonchev Str., bl. 9, 1113 Sofia, Bulgaria

Secondary metabolites are organic molecules of low molecular weight, biosynthesized by any living being using a wide range of biosynthetic pathways, known as secondary metabolism. In evolutionary terms, secondary metabolism is seen as a set of specialized pathways that use a varied and specialized series of enzymes. Secondary metabolism aims to produce molecules with specific functions that promote the adaptability and survival of the species. However, secondary metabolites are not molecules essential to life, as are the lipids, carbohydrates and amino acids involved in basic life functions and produced by the primary metabolism. Terrestrial plants and algae, because they are sessile species, synthesize an admirable structural diversity of secondary metabolites.

Scientific research dedicated to the isolation and identification of secondary metabolites and the evaluation of their potential in different applications has already shown that these small molecules can be used in the promotion of human well-being, in the development of more sustainable agriculture and in the preservation of the environment.

Despite the knowledge already acquired, the consumer looks to the species used in cuisine, as a staple food, spice, or drinks, firstly based on its nutritional value (linked mainly to the primary metabolites present). The consumer is less focused on the different benefits related to the presence of secondary metabolites, such as the effect on the preservation and improvement of the organoleptic quality of the food, their medicinal and cosmetic effects, and as environmentally friendly herbicidal and pesticide agents.

Additionally, the research in this field is by no means complete. There are still many species, edible or not, whose profile in secondary metabolites is unknown and many other whose potential benefits to humanity have not yet been explored; and those in which some applications are already known, but the most relevant probably remains to be determined.

Thus, it remains pertinent to deepen the investigation on the isolation and identification of bioactive secondary metabolites, contributing so that the secondary metabolites present in a species are seen as an asset of that species, expanding the field of applications of the secondary metabolites and the species themselves, valuing them.

The special issue thematic “Isolation and Identification of Bioactive Secondary Metabolites” contributes to look at species beyond their nutritional value, giving them a wider and more efficient use, contributing to more sustainable management of natural resources.

This special issue brings together 12 original research papers which mainly demonstrate the value of several species, based on their secondary metabolites’ composition, and based also in the effect of these metabolites on various applications.

Three of these research papers are dedicated to the identification and evaluation of secondary metabolites with potential pharmacological application.

Celastrol and pristimerin are two quinone-methide triterpenes isolated from species of Celastraceae family, which exhibit various biological activities such as cytotoxic, anti-obesity, and antidiabetic. In addition to the already known biological activities of these two natural compounds present in many species consumed in different world regions for their stimulating properties, Padilla-Montaño et al. [1] demonstrate for the first time the superior antibacterial effect of celastrol against *Bacillus subtilis*, when compared to pristimerin activity. Furthermore, they clarify the action mechanism of celastrol and its ability to act on multiple targets.

Rimpelová et al. [2] describe the cytotoxic effect of two cardiac glycosides, hyrcanoside and deglucohyrcanoside, isolated from seeds of *Coronilla varia* L. (this name is a synonym of *Securigera varia* (L.) Lassen) and reveal the high HEK 293T (transformed kidney cells) cell selectivity of deglucohyrcanoside, better than the well-known anticancer drug digitoxin.

Plants are a source of secondary metabolites that can act as antileishmanicidal agents. An example of this is the family of alkamides, characteristic constituents of *Piper* species that are widely used as culinary spices. (*E*)-Piplartine and (*E*)-demethoxypiplartine, two alkamides isolated from the leaves of *Piper pseudoarboreum* Yunck. exhibit higher potency against *Leishmania* spp. promastigotes and against *Leishmania amazonensis* amastigotes than the reference miltefosine. The activity of (*E*)-piplartine against cutaneous leishmaniasis is demonstrated in an in vivo model [3].

The beneficial health effects demonstrated by the pure secondary metabolites present in plants consumed in any region of the globe, promote these plants in addition to their possible nutritional value. Likewise, the health effects of semi-pure fractions from plant extracts chemically characterized may contribute to increase the value of these species.

The anthocyanins are a group of flavonoid derivatives which exhibit a broad range of bioactivities, such as antioxidant, antimicrobial and anti-inflammatory, and they can cross the blood-brain barrier acting on neurodegenerative targets. Wen et al. [4] present an optimized method for isolation of a rich-anthocyanins fraction from *Aronia melanocarpa* (Michx.) Elliott edible fruits, mainly constituted by four cyanidin 3-*O*-glycosides, and demonstrate, using an in vivo model, the neuroprotective effect of this fraction by improve spatial memory and protect against amyloid-β toxicity.

Compared with olive oil, the chemical composition and potential beneficial effects of olive leaves are still poorly investigated. Taamalli et al. [5] study the chemical composition of supercritical CO_2_ extracts from fresh and dried olive leaves and their effect on hepatotoxicity caused by CCl_4_ in a rat model. The authors identify and quantify 16 compounds (phenolic and terpenoid compounds) on the extracts being their composition significantly distinct. The in vivo study shows the hepatoprotective effect of both olive leaves extracts, due to significant improvement in hepatic fibrosis, biochemical parameters, and oxidative stress level after CCl_4_-induced liver damage.

The edible infusion of bark from *Lycium minutifolium* J. Remy is used in Chilean traditional medicine to treat especially gastrointestinal disorders. This infusion and the ethyl acetate extract were studied by Rodriguez et al. [6] to identify the major secondary metabolites constituents (phenolic acids, flavonoids, coumarins, tropane and spermine alkaloids) and to obtain scientific data on the beneficial effect of these extracts, using an in vivo model and elucidating the action mechanism, to support its known gastroprotective properties. The edible infusion, at 100 mg/Kg, exhibit gastroprotective effect, on HCl/EtOH-induced gastric lesions in mice, higher than the ethyl acetate extract at same dose, and similar to the positive control at 30 mg/Kg.

Secondary metabolites biosynthesized by plant should not only be seen as potential pharmacological agents. Hossen et al. [7] demonstrate the allelopathic effect of *Wedelia chinensis* (Osbeck). Extracts of *Wedelia chinensis* exhibited high inhibitory activity against the root and shoot growth of cress, alfalfa, rapeseed, lettuce, foxtail fescue, Italian ryegrass, timothy, and barnyard grass and could be used for the biological control of weeds. Vanillic acid and gallic acid, isolated from the aqueous methanol extracts significantly arrested the growth of cress and Italian ryegrass seedlings. The concentrations of vanillic acid and gallic acid needed for 50% inhibition (I_50_ values) of the seedling growth of the cress and Italian ryegrass were 0.04–15.4 and 0.45–6.6 mM, respectively.

Several of the published articles on this special issue, in addition to identifying secondary metabolites, evaluate the variation of their content according to the cultivation conditions, between varieties and between different morphological parts of plant.

Hernández et al. [8] describe the phenolic, carotenoid and chlorophyll profile of lamb’s lettuce (*Valerianella locusta* L. Laterr.), a vegetable used in various salads. LC/MS/MS analysis led to identification of 35 phenolic compounds including hydroxybenzoic and hydroxycinnamic acids, flavanols and flavanones. *β*-Carotene and lutein were the major carotenoids. It has also been found that different fertilization doses and salinity levels affected the concentrations of some compounds. The obtained results highlight the importance of these factors on the final metabolites contents of lamb’s lettuce.

In another study, the differences between two subspecies: *Origanum vulgare* L. subsp. *hirtum* (Link) Ietsw. (Greek oregano) and *Origanum* *vulgare* L. subsp. *vulgare* (common oregano) growing in cultivation conditions within temperate climate of Central Europe were examined [9]. Greek oregano was distinguished by visibly higher number of glandular trichomes on the leaves and higher content of essential oil, total phenolic acids and rosmarinic acid in comparison to common oregano. Variation in the content of essential oil and rosmarinic acid in different stages of plant’s development was also observed. The results of this study revealed that Greek oregano could be successfully adapted to different climatic conditions keeping essential oils with a chemical profile typical of Mediterranean cultivars.

In turn, Malarz et al. [10] study chemical constituents of the leaves from the old cultivar of asparagus lettuce (*Lactuca sativa* var. *angustana* cv. Grüner Stern) and compare them with those existing in other lettuce varieties, including wild lettuce. HPLC/DAD and ^1^H NMR analysis of the methanolic extract of asparagus lettuce led to identification of five apocarotenoids, three sesquiterpene lactones, two lignans, five caffeic acid derivatives, and three flavonoids, some of which reported now for the first time in *L. sativa*. Stems, leaves and shoot tips of this variety were also examined to assess their phenolics and sesquiterpene lactone content, as well as DPPH scavenging activity. The results suggest that the leaves of the plant are the richest in antioxidant compounds. The investigated plant material, in terms of polyphenolic content and antioxidative activity, was similar to modern leafy cultivars of *L. sativa*.

Colored tubers of *Solanum tuberosum* L. are increasingly used in food, especially for their appealing colors. The contents of phenolic compounds and anthocyanins, well known for their antioxidant properties, as well as the content of sugars and minerals, were evaluated by Saar-Reismaa et al. [11] in the variety Blue Congo and its crossbreeds of Desiree and Granola and yellow-fleshed tubers. The results show that the content of antioxidant compounds varies among varieties and genotypes, being higher in purple-fleshed tubers than in yellow-fleshed ones. Moreover, the sugar content varies significantly between the studied specimens, with this content showing a positive correlation with the content of anthocyanins.

It is well-known that the profile of secondary metabolites varies depending on the morphological part of the plant. For species used in traditional medicine and gastronomy, it is imperative to know, in detail, the quantitative and qualitative composition of secondary metabolites in each morphological part. Only then will it be possible to take advantage of the potential of each part. Costa et al. [12] study the leaves and rhizome of *Aglaomorpha quercifolia* (L.) Hovenkamp & S. Linds (this name is currently considered synonymous with the Latin binominal name *Drynaria quercifolia* (L.) J.Sm.) used in Timor East in gastronomy and traditional medicine. The results show that the leaves are richest in fatty acids with high nutritional impact (ω6/ω3 ratio, atherogenicity index and thrombogenicity index), whereas the rhizome is richest in terpenes and steroids, some of them with proven medicinal properties.

This special issue also includes three literature review papers that emphasize the added value that the secondary metabolites, and the species they are isolated from, have in the development of new medicines to prevent/treat prevalent diseases.

Jiang et al. [13] present a comprehensive literature revision of the secondary metabolites present in plant-based functional foods that exhibit the ability to lower the level of uric acid, using in vitro and in vivo models. A detailed discussion concerning the targets and the action mechanism associated to their hypouricemic effect is presented.

Leuci et al. [14] review the most recent studies on the isolation and identification of secondary metabolites from plants and fungi, with impactful effects, in vitro and in vivo models, on targets related to the development of cardiovascular and neurodegenerative diseases.

The third review paper [15] focuses on edible species of the botanical genus *Artemisia*. The uses of these species in culinary and beverages and their nutritional value are reviewed, as well as some of their secondary metabolites belonging to the sesquiterpene lactone family. The pharmacological potential and possible adverse effects of these secondary metabolites and species are discussed based on results obtained in in vivo studies and clinical trials.

In conclusion, the fifteen articles published in this special edition reflect the latest research trends regarding the isolation, identification, and assessment of the beneficial effects of secondary metabolites, from edible or inedible species, contributing to these compounds and the plants of origin to be valued beyond the nutritional perspective.
